# Epidemiological Features and Predictors of Mortality in Patients with COVID-19 with and without Underlying Hypertension

**DOI:** 10.1155/2021/7427500

**Published:** 2021-10-19

**Authors:** Leila Moftakhar, Elahe Piraee, Mohammad Mohammadi Abnavi, Parisa Moftakhar, Habibollah Azarbakhsh, Aliasghar Valipour

**Affiliations:** ^1^Abadan University of Medical Sciences, Abadan, Iran; ^2^Student Research Committee, Shiraz University of Medical Sciences, Shiraz, Iran; ^3^Department of Clinical Sciences, Faculty of Veterinary Medicine, Shahid Chamran University of Ahvaz, Ahvaz, Iran; ^4^Department of Public Health, Abadan Faculty of Medical Sciences, Abadan, Iran

## Abstract

**Backgrounds:**

Individuals with hypertension are at higher risk of COVID-19 infection and related mortality. This study was carried out to assess the epidemiological features and predictors of mortality in patients with COVID-19 with hypertension.

**Methods:**

In this retrospective study, the epidemiological characteristics of two groups of patients with COVID-19 with hypertension (1927) and without hypertension (39030) were compared. Chi-square test was applied to evaluate the differences between qualitative variables in two study groups. Logistic regression was also used to determine predictors of mortality in patients with COVID-19 and in patients with COVID-19 with hypertension.

**Results:**

The prevalence of hypertension in patients with COVID-19 was 4.7%, and 24.37% of COVID-19 related deaths occurred in these individuals. The average age of hypertension and nonhypertension patients was 61 and 37 years, respectively. Fever, cough, headache, anorexia, fatigue, and comorbid diseases, such as cardiovascular disease, chronic lung and kidney disease, diabetes, immunodeficiency disease, and thyroid disease, were significantly more frequent in people with hypertension than those without hypertension. The chances of mortality in patient with COVID-19 were 1.8 times higher in individuals with dyspnea, 1.25 in individuals with fever, 1.33 in individuals with cough, 3.6 in patients with hypertension, 2.21 in diabetics, and 2.2 in individuals with cardiovascular disease. Also, individuals with COVID-19 with hypertension that had dyspnea, immunodeficiency, and cardiovascular disease were at higher risk of mortality.

**Conclusion:**

Hypertension is a serious threat to patients with COVID-19. Therefore, in order to control these patients more precisely and reduce mortality in them, it is extremely important to develop prevention and treatment strategies.

## 1. Introduction

Coronavirus disease (COVID-19) is an infectious disease caused by a newly discovered coronavirus. According to the World Health Organization, COVID-19 disease was first reported in December 2019 in Wuhan, China, and rapidly spread across other parts of the world, causing people various health, economic, social, and political problems around the world [1–3]. According to the latest statistics of the World Health Organization, as of April 21, 2021, there are 144,167,456 cases of infection and about 3 million deaths in the world. Also in Iran, 2.31 million people have been infected and 67,000 deaths have occurred. [4].

Recent studies have shown that COVID-19 patients with older age and comorbidity, such as cardiovascular disease, diabetes, hypertension, or chronic lung disease, are at higher risk of infection with a higher mortality rate compared to the general population [5, 6]. In the literature, the prevalence of hypertension in people with COVID-19 was 25.4% in Africa, 31% in China, 49% in Italy, 21% in India, 32% in Oman, and 10% in Iran [7–10]. Therefore, these patients should be given special attention during the epidemic. In another study, the mortality rate of COVID-19 was reported as 6% in patients with hypertension [11].

Given that hypertension is an important cause of mortality in the world, and a large number of people have the disease in the world, during the COVID-19 pandemic, this vulnerable group of people who are at higher risk of morbidity and mortality from COVID-19 should be given more care. For this purpose, the present study aimed to compare the epidemiological features and predictors of mortality in patients with COVID-19 with hypertension.

## 2. Methods

This retrospective study was conducted on 40957 patients with COVID-19 who were admitted in hospitals under the auspices of Abadan University of Medical Sciences in the southwest of Khuzestan province. The total population of this region was estimated as 627970 people, using the databases of the health centers and the national census conducted in 2020. All patients with COVID-19 from March 1, 2019, to March 1, 2020, were enrolled into this study. Confirmation of definite cases of COVID-19 was performed using nasal and throat swab samples (RT-PCR) or by CT scan imaging. Patients with normal vital signs (blood pressure, respiration rate, and pulse) with saturated oxygen levels above 93% and mild symptoms were sent to home quarantine and monitored daily by health professionals. Patients were hospitalized with severe symptoms such as chest pain, shortness of breath, and a saturated oxygen level of less than 90%. Patients with saturated oxygen levels between 90% and 93% were admitted to hospital or undergoing home quarantine. In the area under study, there were only two hospitals that performed all stages of testing and hospitalization of patients according to the national guidelines of corona management, like other medical centers in the country. Clinical and demographic information of patients, and the final outcome of each patient, was recorded.

Duplicate items were identified and removed based on the National ID card. The subjects were divided into two groups of patients, hypertension (*n* = 1927 patients) and nonhypertension (*n* = 39030 patients). Individuals with systolic blood pressure above 14 mmHg and diastolic above 90 mmHg or individuals who take antihypertensive drugs were identified as patients with hypertension. The history of hypertension and other underlying diseases evaluated in this study is based on patients' self-report and questioning the names of their medications.

Variables used in this study include age, gender, final outcome (e.g., death and recovery) and symptoms (e.g., shortness of breath, fever, cough, taste disturbance, fatigue, muscle pain, sore throat, headache, and comorbidities, such as diabetes, cardiovascular disease, kidney and liver diseases, immune deficiency, chronic lung disease, and thyroid disease).

### 2.1. Statistical Analysis

We first described qualitative variables with numbers and percentages and quantitative variables with median. Then, we used the Chi-square test for qualitative variables and the *t*-test for the quantitative variable of age to assess the difference between the mean surviving and not surviving in patients with COVID-19 with underlying hypertension. The normality of the age variable was evaluated by Kolmogorov-Smirnov test.

Finally, we used simple and multiple logistic regression tests to identify the role each of the underlying diseases and other variables on the survival and nonsurvival of patients with COVID-19.

We also used one separately simple and multiple logistic regression tests to identify the factors affecting the mortality of patients with COVID-19 with underlying hypertension.

All variables with P.V less than 0.2 in simple logistic regression test entered to multiple logistic regressions. Significance level was considered 0.05 in all tests. All statistical analyses were performed with SPSS software version 20 and STATA version 14.

## 3. Results

During the study period, 40957 cases of COVID-19 occurred in the cities under the auspices of Abadan University of Medical Sciences in the southwest of Khuzestan province. The median age of the patients was 37 years (IQR 29–50). The majority of patients were male (57.4%), and 26,252 patients (64.01%) had a history of contact with definite or suspected cases of COVID-19. In addition, 1038 patients (2.5%) died, with 24.37% of them having occurred in patients with hypertension (Table 1).

The most common symptoms were fever (59.6%), cough (43.42%), shortness of breath (29.86%), muscle pain (14.52%), and sore throat (7.01%). The most common comorbidities in all study patients include diabetes (6.94%), CVDs (4.88%), chronic lung disease and allergies (2.41%), chronic kidney disease (1.43%), and immunodeficiency diseases (0.95%) (Table 1).

Patients with hypertension accounted for 4.7% of the total study population. Besides, 59.5% of patients with hypertension were female, and 71.35% (*n* = 1375) had a history of contact with definite or suspected cases of COVID-19. Patients with hypertension were older compared to patients without hypertension. The median age of hypertensive patients was 61 years (IQR: 70–50) versus 37 years (IQR: 50–29) in nonhypertensive patients. Furthermore, prevalence of comorbidities was shown to be higher in hypertensive patients, as diabetes (828 (43%)) vs. 2014 (5.16), chronic lung disease (87 (4.5%) vs. 901 (2.3%)), chronic kidney disease (124 (6.4%) vs. 464 (1.19%), systemic disease Immunity (59 (3.06%) vs. 330 (0.84%)), thyroid disease (101 (5.24%) vs. 74 (0.19)), and CVDs (421 (21.84%) vs. 1577 (4.04%)).

Fever, cough, headache, anorexia, and fatigue were significantly higher in hypertensive patients than in nonhypertensive patients. A loss of sense of taste and smell in patients with normal blood pressure was significantly greater in patients with hypertension (Table 1).

The age of patients with COVID-19 with hypertension who died was significantly higher than the age of those who survived (the median age of 68 years (IQR 60–78) vs. 60 years (IQR 49–69)). Fever, cough, and shortness of breath as well as comorbidities, including heart disease, diabetes, and immunodeficiency, as well as history of contact with affected people and hospitalization in the intensive care unit, were significantly higher in those who died than the survivors (Table 2).

The results of logistic regression to determine the predictors of death in all patients with COVID-19 in our study showed that the chance of death is 1.8 times higher in people with dyspnea (OR = 1.82, 95%CI: 1.086–2.206), 1.2 times in people with fever (OR = 1.25, 95%CI: 1.019–1.538), 1.3 times in people with cough (OR = 1.33, 95%CI: 1.101–1.615), 3.6 times in people with hypertension (OR = 3.636, 95%CI: 2.977–4.440), 2.2 times in people with diabetes (OR = 2.215, 95%CI: 1.832–2.676), and 2.2 times in people with cardiovascular disease (OR = 2.237, 95%CI: 1.823–2.745) higher than people without theses sign and disease. Also, the role of age was borderline (OR = 1.002, 95%CI: 1.001–1.003). (Table 3).

The results of logistic regression to determine the predictors of death in all patients with COVID-19 with hypertension showed that the chance of death is 2.04 time in people with dyspnea (OR = 2.046, 95%CI: 1.506–2.779), 2.7 times in people with immunodeficiency (OR = 2.709, 95%CI: 1.424–5.152), and 1.4 times in people with Cardiovascular disease (OR = 1.417, 95%CI: 1.011–1.986) higher than people without theses sign and disease (Table 4).

## 4. Discussion

The present study was performed on 40957 patients with COVID-19 in the southern region of Iran and aimed to compare the clinical features and the rate of mortality among COVID-19 patients with hypertension compared with those patients with normal hypertension. COVID-19 is a highly contagious disease that has spread rapidly around the world [12]. Several studies have shown that although COVID-19 can affect all healthy people of any age, older people and people with chronic disease are at higher risk, and many studies have shown that patients with hypertension are at higher risk for COVID-19 [13–18]. There are also more complications from COVID-19 and hospitalization in the intensive care unit in patient with hypertension [17–19].

In our study, out of the 40,957 surveyed people, 1927 (4.7%) had hypertension. Other studies have reported hypertension as one of the most common chronic diseases in patients with COVID-19 [12, 20–22]. Peng and colleagues reported the prevalence of hypertension among COVID-19 patients as 31% [17]. This rate reported 30% by Zhou and colleagues [12], and 15% by Guan and colleagues [23], while China Centers for Disease Control and prevention revealed this as 13% [24]. In other studies, the prevalence of hypertension in COVID-19 patients has been reported differently as 30.7% [25], 30% [26], and 31.8% [27]. It has been estimated in many studies that people with hypertension make up about one-third of COVID-19 patients, which was shown as about 4.7% in our study. This difference may be due to the very large sample size of our study. Also, about 60% of our study participants aged under 40 years old, which usually had a lower prevalence of underlying diseases at this age. However, some studies focused only on severe forms of the disease or only hospitalized patients in intensive care units and have shown that these people are also more likely to suffer from underlying diseases, such as hypertension.

Many studies have shown that people with hypertension tend to have more severe forms of COVID-19 and are more likely to be hospitalized in the ICU than people without high blood pressure, with a higher rate of mortality [28, 29]. In our study, the rate of mortality in people with hypertension compared to people with normal blood pressure was 1.5% vs. 0.19%. In general, about a quarter (24.3%) of COVID-19 related deaths occurred in hypertensive patients. A systematic review study also found that people with hypertension were 2.2 times more likely to die. [30]. In Spain, the odds of mortality were estimated as 4.7 times [31]. Yang et al. showed the rate of death due to COVID-19 in hypertensive patients as 17.7% [18]. Also, Zhou and colleagues showed that the crude mortality rate was 19.4% in the hypertensive group vs. 2.4% in nonhypertensive group of patients [32], whereas this was reported as 37.1% vs. 8.7% in Cheng's study [18].

Also, the rate of ICU stay in patients with hypertension was 13.13% versus 1.7% in people without hypertension. A systematic review study found that patients with hypertension were 2.1 times more likely to be admitted to the ICU and 1.7 times more likely to have more severe disease [30]. In a meta-analysis study, the odds of admitted to the ICU were estimated to be 2.5 [33]. Also, this rate in people with high blood pressure compared to people with normal blood pressure was estimated 18.6% vs. 8.7% [27] and 32.4% vs. 19.1% [17]. However, Yang and colleagues stated that hypertension was not associated with higher risk of developing severe form of COVID-19 disease [18]. The main reason for the higher mortality rate and hospitalization in the ICU in this group of patients is unknown. However, some studies have suggested the presence of ACE2A enzyme in the body as a possible cause of higher rates of death and ICU stay. This is an internal membrane glycoprotein lung tissue that is found in epithelial cells of the heart, liver, and lungs [34, 35], converts angiotensin 2 to angiotensin 1–7, and increases the anti-inflammatory and antioxidant role of angiotensin 1–7, that leading to vasodilation and lowered blood pressure [36, 37]. Coronavirus also uses ACE2 as a receptor to bind and enter the host body [7, 38–40], thus increasing the expression of ACE2 gene and increasing the risk of COVID-19 infection [41, 42]. ACE2 enzyme is increased in patients with hypertension due to the use of ARBS and ACEIS drugs, which in turn increases the risk of COVID-19 infection. In other studies, hypertension has been suggested to cause pathogenic changes in the cardiovascular system, such as fibrosis and left ventricular hypertrophy, which may increase the odds of getting severe form of the infection due to shortness of breath [43].

The results of our study showed that the chance of death is 2.7 times higher in individuals with impaired immune systems. Impaired immune system is also one of factors associated with the severity of the disease in these people [44]. Some studies have also shown that cytokine storms, such as elevated levels of interleukin-6, interleukin-7, and granulocyte-macrophage cloning replicator, are associated with more severe forms of COVID-19 in people with hypertension [40]. Individuals with hypertension are usually older and often have one or more underlying disease, in which their role in determining the severity of the disease cannot be ignored. In our study, 4.7% of COVID-19 patients had hypertension that 42.6% of them had diabetes and 21.5% had heart disease. Other previous studies have shown that COVID-19 patients with hypertension display higher frequency of different comorbidities [27]. Cheng and et al. stated that 22.9% of hypertensive patients had diabetes versus 4% in nonhypertensive [27]. This may be due to the fact that the proportion of patients with heart problems is higher in people with hypertension [45], and many other studies have found that heart disease and diabetes are much higher in COVID-19 patients with hypertension than in nonhypertensive persons. [29, 32]. Therefore, clinical research is needed to differentiate the role of each factor.

According to the results of the present study, patients with COVID-19 who had hypertension were older than those with normal hypertension (61 years vs. 37 years). This median age has been reported in other studies as follows: in China 69 vs. 59 years [46], in a cohort study in China 59 vs. 41 years [29], and in Huang et al.'s study 67 vs. 57 years [40]. This may be due to the fact that blood pressure usually increases in older age.

In our study, about 60% of COVID-19 cases who had hypertension were males. In another study, it was shown that men were more susceptible to the disease and severe complications, 56.1% [23] and 69.3% [40], which, of course, should be noted that that men are more at risk of developing COVID-19 infection than women, because they leave home more frequently to work and do housework.

The results of our study also showed that the symptoms such as cough, headache, anorexia, and fatigue in hypertensive patients were significantly higher than nonhypertensive COVID-19 patients. Also, the chance of mortality in patients with dyspnea was 2 times. In another study, nocturnal sweating and shortness of breath were shown to be more common in people with hypertension pressure than nonhypertensive [32]. These symptoms can result from the more severe and worsening of the condition of hypertensive patients, who are more severely affected by the disease.

Along with all the topics mentioned, it should be noted that other factors such as environmental factors can play a role in the severity and mortality of COVID-19. The results of a meta-analysis conducted in European countries and China showed that the odds of mortality in people with COVID-19 between March to May are 0.98 times higher per day after admission and 0.85 times higher with increase in temperature of 1°C. Another analysis of the symptoms of 37,184 patients in the UK showed that, during this time, the severity of the disease and the symptoms of the disease decreased [47]. These results indicate the role of environmental factors. Although we did not have data on these factors in our study, we should not ignore their role.

## 5. Limitations

Our study is one of the first in Iran that examined the clinical features of COVID-19 patients with and without hypertension with a relatively large sample size. But one of the limitations of our study was the lack of access to information about the severity of the disease, including the percentage of saturated oxygen and the degree of fever. Therefore, we could not compare the rate of mortality according to the severity of the disease. However, it should be considered that the laboratory and radiological results of patients were not available for more detailed analysis.

## 6. Conclusion

According to the results of our study, hypertension is a major concern for patients with COVID-19, because it increases the rate of hospitalization and death. There is still a lot unknown about COVID-19 disease, and the link between hypertension and COVID-19 that creates great challenges for management and monitoring of these patients for health policymakers. Therefore, in order to control these patients more precisely and effectively, and to reduce the incidence and morbidity, it is recommended to develop prevention and treatment strategies for this group of people who are a vulnerable group against COVID-19. In addition, patients should take preventive measures, use home quarantine, frequently wash their hands, and use blood pressure monitors and controls at home to reduce the number of visits to medical centers.

## Figures and Tables

**Table 1:** The characteristics of hypertensive and nonhypertensive patients with COVID-19.

Variable	Total (*n* = 40957)	Hypertension (*n* = 1927)	Nonhypertension (*n* = 39030)	*P* value
	Number (%)	Number (%)	Number (%)	
Age, median (IQR)	37 (29–50)	37 (29–50)	61 (50–70)	<0.001
Sex				
Male	23521 (57.42)	781 (40.50)	22740 (58.26)	<0.003
Female	17436 (42.58)	1146 (59.50)	16290 (41.74)	
No-sign	1753 (5.50)	94 (4.90)	1659 (4.25)	0.130
Fever	20717 (50.58)	1163 (60.35)	19554 (50.09)	<0.001
Cough	17787 (43.43)	1032 (53.55)	16755 (42.92)	<0.007
Dyspnea	12231 (29.86)	782 (40.58)	11449 (29.39)	0.100
Muscular pain	5948 (14.52)	391 (20.29)	5557 (14.23)	0.100
Sore throat	2872 (7.01)	187 (9.70)	2685 (6.88)	0.350
Anorexia	273 (0.57)	59 (3.10)	214 (0.55)	<0.001
Headache	1263 (3.08)	170 (8.82)	1093 (2.80)	<0.001
Fatigue	313 (0.76)	92 (4.77)	221 (0.56)	0.001
Decreased sense of smell, taste	2176 (5.31)	55 (2.85)	2121 (5.43)	<0.001
Hypoxia	2873 (7.01)	194 (10.07)	2679 (6.86)	0.136
Comorbidities				
Cardiovascular	1998 (4.88)	421 (21.84)	1577 (4.04)	<0.001
Diabetes	2842 (6.94)	828 (43)	2014 (5.16)	<0.001
Immunodeficiency	389 (0.95)	59 (3.06)	330 (0.84)	<0.001
Liver	101 (0.25)	10 (0.52)	91 (0.23)	0.090
Thyroid	175 (0.43)	101 (5.24)	74 (0.19)	0.001
Chronic kidney disease	588 (1.43)	124 (6.43)	464 (1.19)	<0.001
Chronic pulmonary disease	988 (2.41)	87 (4.50)	901 (2.30)	<0.001
Hospitalization history in ICU	102 (0.25)	29 (1.50)	73 (0.19)	<0.001
Exposure to disease	26252 (64.10)	1375 (71.35)	24877 (63.74)	<0.001
Mortality	925 (2.26)	253 (13.13)	672 (1.72)	<0.001

**Table 2:** The baseline characteristics of survivors and nonsurvivors in patients with COVID-19 with hypertension disease.

Variable	Total (*n* = 1927)	Survivors (*n* = 1674	Nonsurvivors (*n* = 253)	*P* value
	Number (%)	Number (%)	Number (%)	
Age, median (IQR)	61 (50–70)	60 (49–69)	68 (60–78)	<0.001

Gender				
Male	781 (40.50)	678 (40.50)	103 (40.71)	0.980
Female	1146 (59.5)	996 (59.50)	150 (59.29)	

Symptoms				
Fever	1156 (59.99)	977 (58.36)	179 (70.75)	0.001
Cough	1028 (53.35)	867 (51.80)	161 (63.64)	0.002
Dyspnea	781 (40.53)	637 (38.05)	144 (56.92)	<0.001
Muscular pain	388 (20.13)	341 (20.37)	47 (18.58)	0.390
Sore throat	185 (9.60)	160 (9.56)	25 (9.88)	0.970
Disguise	54 (2.80)	54 (3.22)	0 (0.00)	0.030
Headache	168 (8.72)	152 (9.08)	16 (6.32)	0.120
Cardiovascular	416 (21.59)	338 (20.20)	78 (30.80)	0.001
Diabetes	822 (42.66)	689 (41.16)	124 (49)	0.04
Immunodeficiency	58 (3.01)	43 (2.57)	15 (5.93)	0.004
Chronic pulmonary disease	87 (4.51)	71 (4.24)	16 (6.32)	0.140
Chronic liver, kidney disease	123 (6.38)	105 (6.27)	18 (7.11)	0.640
Exposure to disease	1357 (78.42)	1195 (71.38)	162 (64.03)	0.010
Hospitalization history in ICU	29 (1.50)	21 (1.25)	8 (3.16)	0.020

**Table 3:** Predictor variables of mortality in patient COVID-19 based on the results of multiple logistic regression.

Predictors	Odds ratio (95%CI)	*P*
Age	1.002 (1.001–1.003)	<0.001
Dyspnea		
No	Reference	
Yes	1.824 (1.086–2.206)	<0.001
Fever		
No	Reference	
Yes	1.252 (1.019–1.538)	0.033
Cough		
No	Reference	
Yes	1.333 (1.101–1.615)	0.003
Hypertension		
No	Reference	
Yes	3.636 (2.977–4.440)	<0.001
Diabetes		
No	Reference	
Yes	2.215 (1.832–2.676)	<0.001
Cardiovascular disease		
No	Reference	
Yes	2.237 (1.823–2.745)	<0.001

**Table 4:** Predictor variables of mortality in COVID-19 patients with hypertension based on the results of multiple logistic regression.

Predictors	Odds ratio (95%CI)	*P*
		
Dyspnea		
No	Reference	
Yes	2.046 (1.506–2.779)	<0.001
Immunodeficiency		
No	Reference	
Yes	2.709 (1.424–5.152)	<0.001
Cardiovascular disease		
No	Reference	
Yes	1.417 (1.011–1.986)	0.043

## Data Availability

The data for the current study will not be shared publicly.
